# Low plasma selenium concentrations in critically ill children: the interaction effect between inflammation and selenium deficiency

**DOI:** 10.1186/cc13877

**Published:** 2014-05-19

**Authors:** Simone Brasil de Oliveira Iglesias, Heitor Pons Leite, Ângela Tavares Paes, Susyane Vieira de Oliveira, Roseli Oselka Saccardo Sarni

**Affiliations:** 1 Department of Pediatrics, Pediatric Intensive Care Unit, Federal University of São Paulo, Rua Loefgreen 1647, 04040-032 São Paulo, SP, Brazil; 2 Department of Pediatrics, Discipline of Nutrition and Metabolism, Federal University of São Paulo, Rua Loefgreen 1647, 04040-032 São Paulo, SP, Brazil; 3 Applied Statistics, Federal University of São Paulo, Rua Loefgreen 1647, 04040-032 São Paulo, SP, Brazil; 4 Department of Pediatrics, Discipline of Allergy and Immunology, Federal University of São Paulo, Rua Loefgreen 1647, 04040-032 São Paulo, SP, Brazil

## Abstract

**Introduction:**

Low plasma selenium concentrations are frequent in critically ill patients. However, whether this is due to systemic inflammation, a deficient nutritional state or both is still not clear. We aimed to determine the factors associated with low plasma selenium in critically ill children while considering the inflammatory response and nutritional status.

**Method:**

A prospective study was conducted in 173 children (median age 34 months) with systemic inflammatory response who had plasma selenium concentrations assessed 48 hours after admission and on the 5th day of ICU stay. The normal reference range was 0.58 μmol/L to 1.6 μmol/L. The outcome variable was ‘low plasma selenium’, which was defined as plasma selenium values below the distribution median during this period. The main explanatory variables were age, malnutrition, sepsis, C-reactive protein (CRP), and clinical severity scores. The data were analyzed using a Binomial Generalized Estimating Equations model, which includes the correlation between admission and 5th day responses.

**Results:**

Malnutrition and CRP were associated with low plasma selenium. The interaction effect between these two variables was significant. When CRP values were less than or equal to 40 mg/L, malnutrition was associated with low plasma selenium levels (odds ratio (OR) = 3.25, 95% confidence interval (CI) 1.39 to 7.63, *P* = 0.007; OR = 2.98, 95% CI 1.26 to 7.06, *P* = 0.013; OR = 2.49, 95% CI 1.01 to 6.17, *P* = 0.049, for CRP = 10, 20 and 40 mg/L, respectively). This effect decreased as CRP concentrations increased and there was loose significance when CRP values were >40 mg/L. Similarly, the effect of CRP on low plasma selenium was significant for well-nourished patients (OR = 1.13; 95% CI 1.06 to 1.22, *P* <0.001) but not for the malnourished (OR = 1.03; 95% CI 0.99 to 1.08, *P* = 0.16).

**Conclusions:**

There is a significant interaction between the magnitude of the inflammatory response and malnutrition on low plasma selenium. This interaction should be considered when interpreting plasma concentrations as an index of selenium status in patients with systemic inflammation as well as in the decision on selenium supplementation.

## Introduction

Selenium plays an essential role in the protection against lipid peroxidation, in regulating T cell activity, mediating the inflammatory response and aiding thyroid hormone metabolism. The biological effects of selenium are achieved by 25 selenoproteins, among which the most known are selenoprotein P, the glutathione peroxidases, thioredoxin reductases and iodothyronine deiodinases [1]. Plasma selenium is predominantly associated with three proteins: selenoprotein P, which comprises over 50% of plasma selenium, glutathione peroxidase and albumin, which accounts for 20 to 40% and 9% of plasma selenium, respectively [2]. Plasma concentration reflects a very low part of body selenium as there is only 0.2 mg selenium in plasma for 20 to 40 mg in the whole body [3].

Decreased plasma concentrations during the acute phase response have been described in animal and clinical studies [4,5]. Plasma selenium is reportedly significantly lower in critically ill adult patients compared with healthy subjects [6-10] and is associated with oxidative stress, infectious complications, organ dysfunction and higher mortality [8,9,11,12]. However, in an international, randomized, blind trial conducted in patients with multiorgan failure, the North American patients did not show the low plasma selenium concentrations consistently observed in European and South American trials of selenium status in critically ill and healthy people. These differences were attributed to the depletion of selenium in soil observed in parts of Europe but not in North America [13]. There are few data about the natural abundance of selenium in Brazilian soils, as well as on the intake of selenium by the population. Evidence of low intake of selenium is reported in São Paulo, an area considered to have a selenium-deficient soil [14,15].

Low plasma selenium concentrations have also been reported in critically ill children [16-18]. However, the extent to which low plasma selenium concentrations reflect systemic inflammation, a selenium-deficient nutritional status or both is still not clear. The assessment of selenium nutritional status by biomarkers should ideally consider the high prevalence of malnutrition in ICUs [19,20] as well as a previous selenium deficiency. Correct analyses of these data are important for nutritional management of the critically ill patient. To date, there are no studies assessing the influence of malnutrition on selenium plasma concentrations in patients with systemic inflammation.

Based on the hypothesis that selenium plasma concentrations in children that are admitted to the ICU are reduced compared with normal standards, especially in malnourished patients, we sought to understand the risk factors that are associated with low plasma selenium concentrations in critically ill children while taking patient nutritional status and the magnitude of the systemic inflammatory response into account.

## Methods

This prospective observational study was conducted in a teaching hospital ICU with level I accreditation [21]. One hundred and seventy-three children who were admitted between July 2009 and May 2011 with systemic inflammatory responses were eligible for inclusion in the study. Neonates were excluded, as well as children with liver or kidney diseases; patients who were expected to be admitted for less than 24 hours, those with encephalic death, and readmissions were also excluded. The study was approved by the Research Ethics Committee of Federal University of São Paulo and written informed consent was obtained from the parents of each patient.

Nutritional therapy was performed according to the ICU protocol and was initiated after nutritional status assessment in the setting of hemodynamic stability. Patients were considered as hemodynamically stable if they were not hypotensive and did not require significant hemodynamic support including high-dose catecholamine agents, alone or in combination with large-volume fluid or blood product resuscitation to maintain cellular perfusion [22]. Feeding was preferably delivered by the enteral route. Energy requirements for the systemic inflammatory response acute phase were calculated according to the predicted basal metabolic rate, initially 50% of the total volume. The infusion rate increased every 12 to 24 hours, if tolerated, to reach 100% of total estimated volume by day two [23].

### Variables

The outcome variable was ‘low plasma selenium’, which was defined as plasma selenium value below the distribution median. The following factors were considered to be exposure variables that could potentially affect the outcome: age, gender, nutritional status upon admission, diagnosis of severe sepsis/septic shock on admission, magnitude of the inflammatory response as measured by serum C-reactive protein (CRP) concentrations, albumin and lactate serum concentrations, clinical severity upon admission and organ dysfunction scores.

#### Laboratory analyses

Blood samples for plasma selenium, CRP, serum albumin, and lactate concentration analysis were obtained at an average time of 48 hours after admission and on the 5^th^ day of ICU stay. Plasma selenium concentration was determined using graphite furnace atomic absorption spectrophotometry with Zeeman background correction. The normal reference range was 0.58 μmol/L to 1.6 μmol/L, which corresponded to the mean values proposed for the healthy pediatric population [24].

Serum CRP concentrations were assessed by turbidimetry [25] on the basis of a reference normality value ≤10 mg/L for acute-phase inflammatory responses. Serum albumin and lactate analyses were performed using the colorimetric method, with normal lactate concentrations defined as ≤2 mMol/L.

#### Nutritional status assessment

For nutritional status classification, the anthropometric indicators weight for age (W/A), height for age (H/A) and body mass index (BMI) were compared against the World Health Organization 2006 growth standards [26]. For children younger than 2 years, we used the W/A or H/A whereas the BMI was used for children older than 2 years of age. Patients with an anthropometric index z score below –2 were considered malnourished. Calculations were performed using the World Health Organization Anthroplus software (version 1.0.2; World Health Organization, Geneva, Switzerland).

#### Clinical assessment

Clinical severity scores on admission were assessed according to the Revised Pediatric Index of Mortality (PIM 2) [27]. The Pediatric Logistic Organ Dysfunction score (PELOD) [28] score was used to assess multiple organ dysfunction severity on the day of admission. Systemic inflammatory response syndrome, severe sepsis, and septic shock were defined according to pediatric consensus terminology [29]. ICU-free days were defined as days not needing ICU care in the first 28 days after admission. Subjects who did not survive to day 28 or who stayed in the ICU for 28 days or more were assigned zero ICU-free days. Ventilator-free days were defined as the number of days alive and breathing without assistance from admission to day 28. Subjects who did not survive to day 28 were assigned zero ventilator-free days [30].

### Statistical analysis

Categorical data were summarized using frequencies and percentages, and normally distributed or non-normal quantitative data were summarized using means and standard deviations or median and interquartile range. Laboratory parameters observed on admission and on the 5^th^ day of ICU stay were compared using a paired *t* test and the Wilcoxon test.

Two approaches were used to evaluate the effect of explanatory variables on the outcome: first, using only admission values and second, with the two measurements (admission and 5th day). In the first approach, logistic regression models were fitted. Generalized estimating equation (GEE) models with binomial distribution were used in the second approach, because they take the dependence observed between two values within a patient into account. Univariate and multivariate analyses were performed in both approaches. Variables with a *P* <0.10 in the univariate models were selected for the multivariate model. The interaction terms among variables, which remained in the final models, were investigated. The significance level was set to 0.05. Intercooled Stata 10.0 (StataCorp LP, College Station, TX, USA) software was used to perform the analysis.

## Results

The main patient characteristics are summarized in Table 1. Blood samples were collected from all patients on admission and from 99 of these patients who remained in the ICU until day 5. Median plasma selenium concentration at admission was 0.29 μmol/L (interquartile range 0.18 to 0.38 μmol/L); values below the lower limit of normal (0.58 μmol/L) were observed in 90.7% (157/173) of patients. Other laboratory values at admission were as follows: the median CRP concentration was 45.1 mg/L (interquartile range 12.5 to 111.9 mg/L), albumin (mean ± SD) was 3.12 ± 0.67 g/dL and median lactate was 1.0 mMol/L (interquartile range 0.8 to 1.6 mMol/L). CRP concentrations decreased from admission to 5^th^ day while the other variables remained relatively constant (Table 2). Selenium concentrations persisted below normal levels in 82 of the 99 patients who remained in the ICU until day 5. The mean daily intake of selenium was 6.8 μg (range 0 to 48.7 μg) and only six patients achieved the Estimated Average Requirement or Adequate Intake for selenium during the study period. In a multivariate model adjusted for age and CRP, selenium intake was not related to a decreased risk of low plasma selenium on day 5

**Table 1:** Main clinic and demographic characteristics of the patients at admission (n = 173)

**Variable**	**Values**
Age* (months)	34 (9-90)
Male gender - n (%)	106 (61)
**Nutritional status - n (%)**	
Well nourished	90 (52.0)
Malnourished	80 (46.3)
Obese/overweight	3 (1.7)
Mortality n (%)	11 (6.3)
PIM 2* 3.68	(1.32 – 8.78)
PELOD (on admission)*	11 (2-12)
ICU-free days (days)*	21 (15-24)
Length of hospital stay (days)*	15 (10-27)
Ventilator-free days (days)*	24 (20-27)
**Diagnostic groups - n (%)**	
Surgical	**77 (44.5)**
Cardiovascular	36 (46.7)
Gastrointestinal	13 (17)
Neurological	11 (14.2)
Miscellaneous	17 (22.1)
Medical	96 (55.5)
Respiratory	70 (73)
Cardiovascular	7 (7.2)
Gastrointestinal	7 (7.2)
Neurological	8 (8.4)
Endocrine/Metabolic	4 (4.2)

*Data expressed as median and interquartile range. PIM 2, revised Pediatric Index of Mortality; PELOD, Pediatric Logistic Organ Dysfunction score.

**Table 2:** **Comparative analysis between laboratory parameters on admission and on the 5**^**th **^**day of ICU stay - patients with at least 5 days of ICU stay (n = 99)**

	**Day of assessment**		
**Variables**	**Admission**	**5th day**	***P *** **value**
Selenium* (μmol/L)	0.30 (0.15-0.39)	0.32 (0.20-0.49)	<0.001^w^
CRP* (mg/L)	49.3 (18.8-112.9)	29.4 (7.0-73.5)	<0.001^w^
Lactate* (mMol/L)	1.0 (0.8-1.5)	1.0 (0.7-1.4)	0.21^w^
Albumin** (g/dL)	3.0 ± 0.61	3.29 ± 0.53	<0.001^t^

*Data expressed as median and interquartile range; **data expressed as the mean and standard deviation; ^W^Wilcoxon test; ^t^paired *t* test. CRP, C-reactive protein.

The outcome ‘low plasma selenium’, defined by plasma selenium concentration below the distribution median (≤0.29 μmol/L on admission and ≤0.32 μmol/L on the 5^th^ day of ICU stay, respectively) was analyzed at the two time points (admission and hospitalization day 5).

### Low plasma selenium on admission

Univariate and multivariate analyses of exposure variables for low plasma selenium at admission are demonstrated in Table 3. Malnutrition and CRP concentrations on admission were statistically associated with the final model outcome.

**Table 3:** Effect of exposure variables on low plasma selenium on admission

	**Univariate analysis**		**Multivariate analysis**	
**Variables**	**OR (95% CI)**	***P value***	**OR (95% CI)**	***P value***
Male gender	0.70 (0.38-1.29)	0.25	-	*-*
Age (months)	0.99 (0.99-1.00)	0.50	-	*-*
PELOD	1.02 (0.98-1.07)	0.25	-	*-*
PIM 2	1.01 (0.99-1.04)	0.24	-	*-*
Surgical	1.07 (0.59-1.05)	0.82	-	
Sepsis/septic shock	2.48 (0.62-9.93)	0.199	-	-
Malnutrition	2.39 (1.30-4.41)	0.005	2.05 (1.07-3.90)	0.03
CRP (x 10 mg/dL)	1.08 (1.03-1.12)	0.001	1.06 (1.01-1.11)	0.025
Albumin (g/dL)	0.63 (0.39-0.99)	0.048	0.88 (0.55-1.49)	0.65
Lactate (mMol/L)	1.00 (0.98-1.03)	0.73	-	-

Univariate and multivariate logistic regression analyses (n = 173). OR, odds ratio*;* CI, confidence interval; PELOD, Pediatric Logistic Organ Dysfunction score; PIM 2, revised Pediatric Index of Mortality; CRP, C-reactive protein.

To better understand the interference of malnutrition and CRP on low plasma selenium, the interaction between these variables was studied and demonstrated to be statistically significant (*P* = 0.035). Interaction between variables is present when the effect of one predictor variable on the outcome varies according to the other independent variable. In our study, the significant interaction suggests that the effect of malnutrition on the outcome (low plasma selenium) depends on CRP concentration and the effect of CRP on the outcome depends on whether the child is malnourished or not. This result will be described in detail in the analysis of the two time points (admission and on day 5).

### Low plasma selenium at the two time points (admission and hospitalization day 5)

Univariate and multivariate analyses of exposure variables for low plasma selenium on admission and day 5 are demonstrated in Table 4. The results are very close to those that were found in the low plasma selenium on admission analysis (Table 3). CRP values on admission and malnutrition were significantly associated with low plasma selenium for both evaluation time points.

**Table 4:** Effect of exposure variables on low plasma selenium (admission and day 5)

	**Univariate analysis**		**Multivariate analysis**	
**Variables**	**OR (CI 95%)**	***P value***	**OR (CI 95%)**	***P value***
Male gender	0.69 (0.39-1.21)	0.19	-	*-*
Age (months)	1.00 (0.99-1.00)	0.11	-	*-*
PELOD	1.03 (0.99-1.07)	0.16	-	*-*
PIM 2	1.17 (0.67-2.04)	0.58	-	*-*
Surgical	1.07 (0.61-1.86)	0.82	-	
Sepsis/septic shock	1.44 (0.43-4.89)	0.55	-	-
Malnutrition	2.04 (1.15-3.62)	0.005	1.88 (1.05-3.39)	0.035
CRP on admission (x 10 mg/L)	1.08 (1.04-1.13)	<0.001	1.06 (1.02-1.11)	0.007
Albumin (g/dL)	0.59 (0.38-0.92)	0.018	0.88 (0.53-1.43)	0.58
Lactate (mMol/L)	1.00 (0.98-1.02)	0.89	-	-

Results of the generalized estimating equations models (*GEE).* OR, odds ratio*;* CI, confidence interval; PELOD, Pediatric Logistic Organ Dysfunction score; PIM 2, revised Pediatric Index of Mortality; CRP, C-reactive protein.

**Figure 1:**
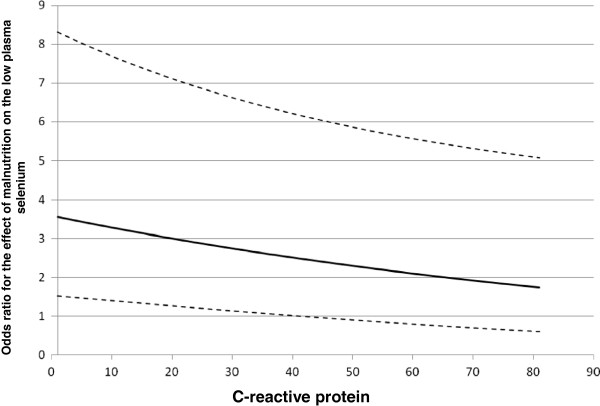
**Effect of malnutrition on low plasma selenium concentrations (odds ratio) according to CRP values during the study period.** The solid line represents the odds ratio; dotted lines represent the 95% confidence interval limits. CRP, C-reactive protein.

Such as for the analysis of the outcome on admission, there was a significant interaction between CRP concentrations and malnutrition (*P* = 0.03). To better evaluate this result, estimates of the effects of these two variables on patient outcome are presented in Table 5. The effect of CRP concentrations on low plasma selenium was significant in well-nourished (*P* <0.001) but not in malnourished patients (*P* = 0.16). The effect of malnutrition on low plasma selenium also decreased according to the CRP concentration increase and was no longer significant from values >40 mg/L (Figure 1). This cutoff value for CRP derived from the statistical interaction between CRP values and malnutrition.

**Table 5:** Effects of CRP concentrations and malnutrition on low plasma selenium

**Effects**	**OR**	**95% CI**	***P***
**Effect of CRP (x 10 mg/L)**			
Well-nourished patients	1.13	1.06-1.22	<0.001
Malnourished patients	1.03	0.99-1.08	0.16
Effect of malnutrition*			
Patients with 10 mg/L CRP	3.25	1.39-7.63	0.007
Patients with 20 mg/L CRP	2.97	1.255-7.06	0.013
Patients with 40 mg/L CRP	2.49	1.01-6.17	0.049
Patients with 60 mg/L CRP	2.09	0.79-5.53	0.14
Patients with 80 mg/L CRP	1.74	0.60-5.07	0.31

*Comparison between malnourished and well-nourished patients regarding the chance of low plasma selenium concentrations; OR, odds ratio*;* CI, confidence interval; CPR, C-reactive protein.

## Discussion

Plasma selenium concentrations were below the normal range in most patients during the study period. Because infants have a high metabolic rate and faster growth, they are expected to represent a high-risk age group for deficiency. Since there are no reference values for plasma selenium in normal Brazilian children, the reference recommended by the laboratory were adopted in our study. The reference values for plasma selenium concentrations must be adjusted for age because serum selenium concentrations in childhood show a significant age dependency [24]. We found three studies in the literature that evaluated plasma selenium levels during the systemic inflammatory response in children that had been admitted to the ICU [16-18]. Plasma selenium concentrations were below the reference values in up to 82% of patients, but no studies considered patient nutritional status and the magnitude of the inflammatory response.

We did not find correlation between plasma selenium and severity of illness indices. In another pediatric study, weak correlations were found between these variables, suggesting that other factors may affect plasma selenium and severity of illness [18].

Plasma concentration analysis is the most commonly used method for selenium determination [31]. However, plasma concentrations of some micronutrients may be affected by systemic inflammation, which may confound the low plasma concentrations, which indicate deficiency [32,33]. The main factor responsible for reduced plasma concentrations during the systemic inflammatory response is, hypothetically, selenium redistribution from the circulation to tissues that are involved in immune function. Endothelial injury results in increased vascular permeability favoring transcapillary escape of selenium transporter proteins [34]. In addition to ischemia-induced local acidosis which causes selenoprotein P binding to the activated endothelium [35,36], acute losses through biological fluids, dialysis, and low intake contribute to low plasma selenium concentrations in critically ill patients [11]. The molecular mechanisms that lead to reduced serum selenium during the acute phase response have been studied in animal models. In a study in mice, the authors demonstrated that genes essential for selenium metabolism are downregulated as part of the acute-phase response and impair regular selenoprotein P biosynthesis by hepatocytes [37]. Experimental data also indicate that kinetics of plasma selenium during the early stages of systemic inflammation are quite similar in mammals [4,5,37,38]. Very early decreases in plasma selenium concentrations have been reported after an endotoxemia-induced acute response in rats [5] and in an ovine model of septic shock [38]. In a study to evaluate the time-dependent changes in plasma trace elements in rats following burn injury, plasma selenium decreased six hours post injury with no detectable changes in tissue selenoenzymes activity [4], suggesting evidence in favor of a conserved tissue selenoenzymes activity during acute inflammation.

Apart from the systemic inflammatory response, selenium deficiency should be understood as a true low selenium status with decreased tissue selenoenzyme activity [39] and that this deficiency leads to low plasma selenium concentration, as well as low selenoprotein P or glutathione peroxidase [40,41]. In addition, considering that selenium content in plasma is a very small part of body selenium content (0.5 to 1%) one cannot deduce from a low plasma selenium concentration a low tissue selenoenzyme activity and thus a true selenium deficiency [3].

Serial serum CRP concentration monitoring may be useful to provide a more accurate interpretation of plasma selenium concentration as a nutritional deficiency indicator. In a recent cross-sectional study on a large blood sample from adult patients with various types of medical conditions, selenium and other micronutrient plasma concentrations decreased with inflammatory response intensity as assessed by CRP. The authors suggested plasma micronutrient concentrations can only be clinically interpreted with knowledge of the degree of inflammatory response. According to their results, plasma selenium concentration assessment would be feasible if CRP values were lower than 10 mg/L [42]. Given that these were the results of routine micronutrient screens that were extracted from the laboratory database, patient clinical and nutritional status was not considered. The studies in which nutritional status was considered a risk factor for low plasma selenium concentrations were all performed in *kwashiorkor*[43], HIV [44,45] and tuberculosis [46] patients, which is different from the children that were admitted to the ICU.We evaluated plasma selenium concentrations in critically ill children taking the magnitude of inflammatory response and nutritional status, among other variables, into account. Both factors were associated with increased low plasma selenium concentration risk. As represented in Figure 1, the effect of malnutrition on low plasma selenium concentrations depended on CRP values. When CRP values were less than or equal to 40 mg/L, malnutrition significantly increased the chance of low plasma selenium. With values higher than 40 mg/L, the magnitude of the inflammatory response prevailed over malnutrition, which ceased to be significantly associated with the outcome. Similarly, there was a significant affect of CRP concentrations on low plasma selenium in well-nourished patients but not in the malnourished. Malnutrition must be considered when understanding low plasma selenium concentrations, especially in patients in which the inflammatory response is not as severe. In this context, malnutrition somewhat reduced the importance of CRP concentrations on low plasma selenium risk. Therefore, one must consider both factors - the magnitude of the inflammatory response as well as nutritional status when interpreting plasma selenium concentrations in patients under metabolic stress.

### Study limitations

The main limitation of this study was defining the low plasma selenium outcome variable based on the median study sample selenium value. In our study, the definition of the median as a cutoff was adopted because, similar to other adult and child studies [6-10,16-18], plasma selenium concentrations were below the lower limit in most patients. This factor coupled with the high prevalence of malnutrition in our ICU plus the evidence of selenium deficiency in some areas of Brazil [14,15], should be considered when generalizing these results to other units with different profile. In addition, the relation between malnutrition and selenium deficiency should be nuanced. Although multiple micronutrient deficiencies are inherent in malnutrition, selenium deficiency may occur without visible protein-energy malnutrition depending on the selenium content of the food. Hence, the link between malnutrition and nutritional selenium deficiency depends on the selenium content of the soil and food [47] and on the type of malnutrition.

It is well established that selenium is an essential micronutrient for antioxidant defenses that has reduced plasma concentrations during a systemic inflammatory response. However, the benefit of selenium supplementation in all critically ill patients is still not proven. Meta-analyses have shown that high-dose selenium might have beneficial effects in patients with sepsis syndrome, but there are significant heterogeneity of protocols, patients and outcomes in the different trials [10,48-51].

The results of our study show that low plasma concentrations are not necessarily indicative of systemic inflammation only, but may also reflect nutritional deficiency. Low plasma selenium concentrations in malnourished patients may be an indication for supplementation, whereas this does not necessarily mean that all patients with low plasma selenium should be supplemented. Importantly, an interpretation that considers only plasma selenium concentrations and omits nutritional status may have implications for nutritional therapy in patients undergoing a systemic inflammatory response. Although there are no reports of adverse effects of selenium supplementation for a short period of time in critically ill patients, it is worth considering that high selenium compound concentrations are toxic and that sodium selenite may also act as an oxidant molecule [3,34,52,53]. Adjusted hazard ratios for serum selenium-induced all-cause mortality among US adults have demonstrated that whereas additional selenium intake may benefit people with low plasma selenium concentrations, those with adequate to high concentrations might be affected adversely and should not be supplemented [1,52]. Supplementation with doses around or below the tolerable upper intake (UL for adults is 400 μg/day) should be sufficient to correct previous selenium deficiency in critically ill patients [34,54].

## Conclusions

In this study, we have shown that the magnitude of the inflammatory response and malnutrition are associated with low plasma selenium in children admitted to the ICU. There is a significant interaction between these two factors on plasma selenium concentrations. This interaction should be considered when interpreting plasma concentrations as an index of selenium status in patients with systemic inflammation as well as in the decision on selenium supplementation.

## Key messages

• There is an interaction between the magnitude of the inflammatory response and malnutrition on low plasma selenium concentrations in critically ill children. Interaction between variables is present when the effect of one predictor variable on the outcome varies according to the other independent variable.

• The effect of malnutrition on low plasma selenium depends on CRP concentrations. At CRP values less than or equal to 40 mg/L malnutrition increased the chance of low plasma selenium. This effect decreased according to the CRP concentration increase and was no longer significant from values >40 mg/L. In turn, the effect of CRP concentrations on low plasma selenium depends on whether the child is malnourished or not. This effect was significant in well-nourished but not in malnourished patients.

• The interaction between CRP and malnutrition should be considered when interpreting plasma concentrations as an index of selenium status in patients with systemic inflammation as well as in the decision on selenium supplementation.

## Abbreviations

BMI: body mass index; CI: confidence interval; CRP: C-reactive protein; GEE: generalized estimating equations; H/A: height for age; HIV: human immunodeficiency virus; ICU: intensive care unit; OR: odds ratio; PELOD: Pediatric Logistic Organ Dysfunction score; PIM 2: revised Pediatric Index of Mortality; W/A: weight for age.

## Competing interests

The authors declare that they have no competing interests.

## Authors’ contributions

The authors’ responsibilities were as follows: SBO participated in the study design, data acquisition and in the manuscript writing and review; HPL planned and designed the study design, participated in the statistical analysis, manuscript writing and review; ATP participated in the statistical analysis and interpretation of data and review; SVO participated in the study design, data acquisition and review, ROS participated in the interpretation of data, manuscript writing and review. All authors read and approved the final manuscript.
